# Differences in Calcium Clearance at Inner Hair Cell Active Zones May Underlie the Difference in Susceptibility to Noise-Induced Cochlea Synaptopathy of C57BL/6J and CBA/CaJ Mice

**DOI:** 10.3389/fcell.2020.635201

**Published:** 2021-02-05

**Authors:** Hongchao Liu, Hu Peng, Longhao Wang, Pengcheng Xu, Zhaoyan Wang, Huihui Liu, Hao Wu

**Affiliations:** ^1^Department of Otolaryngology-Head and Neck Surgery, Shanghai Ninth People's Hospital, Shanghai Jiao Tong University School of Medicine, Shanghai, China; ^2^Ear Institute, Shanghai Jiao Tong University School of Medicine, Shanghai, China; ^3^Shanghai Key Laboratory of Translational Medicine on Ear and Nose Diseases, Shanghai, China; ^4^Department of Otolaryngology-Head and Neck Surgery, Changzheng Hospital, Second Military Medical University, Shanghai, China

**Keywords:** noise-induced cochlear synaptopathy, calcium clearance, mitochondrial, inner hair cell, presynaptic ribbon

## Abstract

Noise exposure of a short period at a moderate level can produce permanent cochlear synaptopathy without seeing lasting changes in audiometric threshold. However, due to the species differences in inner hair cell (IHC) calcium current that we have recently discovered, the susceptibility to noise exposure may vary, thereby impact outcomes of noise exposure. In this study, we investigate the consequences of noise exposure in the two commonly used animal models in hearing research, CBA/CaJ (CBA) and C57BL/6J (B6) mice, focusing on the functional changes of cochlear IHCs. In the CBA mice, moderate noise exposure resulted in a typical fully recovered audiometric threshold but a reduced wave I amplitude of auditory brainstem responses. In contrast, both auditory brainstem response threshold and wave I amplitude fully recovered in B6 mice at 2 weeks after noise exposure. Confocal microscopy observations found that ribbon synapses of IHCs recovered in B6 mice but not in CBA mice. To further characterize the molecular mechanism underlying these different phenotypes in synaptopathy, we compared the ratio of Bax/Bcl-2 with the expression of cytochrome-C and found increased activity in CBA mice after noise exposure. Under whole-cell patch clamped IHCs, we acquired two-photon calcium imaging around the active zone to evaluate the Ca^2+^ clearance rate and found that CBA mice have a slower calcium clearance rate. Our results indicated that excessive accumulation of calcium due to acoustic overexposure and slow clearance around the presynaptic ribbon might lead to disruption of calcium homeostasis, followed by mitochondrial dysfunction of IHCs that cause susceptibility of noise-induced cochlear synaptopathy in CBA mice.

## Introduction

Hearing loss is one of the most common health problems that affects communication and impacts life quality (Lin et al., 2011). Hearing loss is often caused by the death of sensory hair cells (HCs) in the inner ear, which function in transducing the sound waves into electric signals (Wang et al., 2017; Zhu et al., 2018; Liu et al., 2019d; Qi et al., 2019, 2020). Damages from a variety of intrinsic and extrinsic sources can impair inner ear function, including mutations in deafness genes, exposure to ototoxic drugs, aging, chronic cochlear infections, and environmental noise overexposure (Kujawa and Liberman, 2009; Gao et al., 2019; Zhang et al., 2019, 2020; He et al., 2020; Qian et al., 2020; Zhong et al., 2020; Zhou et al., 2020), among which, noise-induced hearing loss (NIHL) is the most common form of non-hereditary sensorineural hearing loss, with the incidence increasing annually in our aging society.

Severe noise exposure can damage cells in the inner ear, resulting in HC loss and elevated hearing thresholds. However, recent studies showed that a moderate level of acoustical exposure might not lead to permanent threshold shift but instead cause permanent synapse loss (Kujawa and Liberman, 2009; Liberman et al., 2016; Liberman and Kujawa, 2017). The inner hair cell (IHC) and spiral ganglion neuron (SGN) synapse, both pre- and postsynaptic structures, are vulnerable to noise exposure (Kujawa and Liberman, 2015; Liu et al., 2019b; Michanski et al., 2019). This cochlear synaptopathy is an “auditory processing disorder” that alters auditory information processing and leads to difficulties in speech discrimination in noisy environments (Shi et al., 2016). CBA/CaJ (CBA) and C57BL/6J (B6) mice are two of the most common animal models used in hearing research (Brewton et al., 2016). In our previous study, we compared differences in cellular functions in the cochleae of B6 and CBA mice and concluded that excessive loading of Ca^2+^ over a prolonged period might damage IHCs (Liu et al., 2019a). Cytosolic calcium homeostasis requires efficient Ca^2+^ clearance through a combination of Ca^2+^ pumps, Ca^2+^ buffers, and intracellular Ca^2+^ stores (Tucker and Fettiplace, 1995; Zenisek and Matthews, 2000; Carafoli, 2011). Presynaptic-Ca^2+^ influx through voltage-gated Ca^2+^ channels initiates mitochondrial-Ca^2+^ uptake around the ribbons and subsequent mitochondrial damage (Wong et al., 2019). Based on such a notion, this study further analyzes the functional alterations of IHC ribbon synapses after one episode of noise exposure and explore possible mechanisms in functional differences between B6 and CBA mice.

Noise-induced synaptopathy precedes the more commonly considered form of sensorineural deafness associated with damage to the outer HCs, which leads to the reduction in auditory nerve innervation to the IHCs (Monaghan et al., 2020). This synaptic degeneration may contribute to the generation of tinnitus, hyperacusis, and associated perceptual abnormalities (Kaltenbach and Afman, 2000; Schaette and McAlpine, 2011; Hickox and Liberman, 2014). Gaining understandings of the possible mechanisms underlying damage to the ribbon synapses is an important step in preventing noise-induced cochlear synaptopathy (Vlajkovic et al., 2017; Wang et al., 2019). Our experiments have revealed that excessive accumulation due to slow clearance of calcium around the presynaptic ribbon during acoustic overexposure may lead to disruption of calcium homeostasis, followed by mitochondrial dysfunction of IHCs that cause susceptibility of noise-induced cochlear synaptopathy in CBA mice.

## Materials and Methods

### Animals

Male CBA and B6 mice aged 3–4 weeks old were obtained from SIPPR-BK Laboratory Animals Ltd. (Shanghai, China). Mice were housed for the duration of the experiments in the animal care facility of Ear Institute of Shanghai 9th people's hospital, in affiliation with Shanghai Jiao Tong University School of Medicine. The experimental procedures described were approved by the University Committee of Laboratory Animals of Shanghai 9th people's hospital and followed the guidelines for the Care and Use of Laboratory Animals (8th edition), published by the National Institutes of Health (Bethesda, MD, USA).

### Hearing Assessment and Acoustic Exposure

As described previously (El-Hassar et al., 2019; Lin et al., 2019), recordings of the auditory brainstem responses (ABRs) were made on an anesthetized animal (chloral hydrate, 480 mg/kg intraperitoneal), and body temperature was maintained at near 37°C throughout recording with a heating blanket (Harvard Apparatus, Holliston, MA, USA; 55-7020). For the recordings, three needle electrodes were placed subdermally at the vertex (active), left mastoid area (reference), and right shoulder (ground). An MF-1 speaker was placed in front of the animal 10 cm away from the vertex. Short tone burst stimuli (3-ms duration, 1-ms rise/fall times) were delivered free field. Stimulus frequencies rove from 32 to 4 kHz in half-octave steps. For each measured frequency, the sound level starts from 90 to 0 dB of sound pressure level (SPL) with 5 dB decrement or until two levels below visible thresholds. Each waveform was averaged 400 times. The hearing thresholds were determined by the minimal stimulus level that evoked any noticeable ABRs at each frequency. Amplitudes (μV) of ABR wave I were measured and exported offline using BioSigRZ software (Tucker-Davis Technologies, Alachua, FL, USA). Amplitude was measured by averaging the ΔV of both sides of the peak (Tan et al., 2017; Zhao et al., 2020).

Noise exposure was induced by exposing conscious animals in a calibrated reverberating chamber where differences in sound pressure level varied ~1 dB in typical locations. Bandpass noise of 2–20 kHz at 103 dB SPL was delivered for 2 h by an amplifier and loudspeaker (Yamaha). Noise signals were generated by a TDT RZ6 system (Tucker-Davis Technologies) and calibrated to the target sound pressure level immediately before each acoustic overexposure by acoustimeter (type AWA6228+, Hangzhou Aihua). ABR recordings were performed before and repeated at 1 and 14 days after noise exposure.

### Two-Photon Ca^2+^ Imaging

Ten mice of each group were used, and the organ of Corti was dissected in the cold extracellular solution containing the following (in millimolar): 115 sodium chloride, 2.8 potassium chloride, 25 tetraethylammonium chloride, 5 calcium chloride, 1 magnesium chloride, 2 sodium pyruvate, 5.6 D-glucose, and 10 4-(2-hydroxyethyl)-1-piperazineethanesulfonic acid (300 mOsm, pH 7.40). For Ca^2+^-imaging, IHCs were whole-cell patch clamped at the apical turn of the basilar membrane; the selected location corresponds to ~8.0 kHz frequency region. An EPC10 amplifier controlled by Patchmaster 10.0 pulse software (HEKA Elektronik, Harvard Bioscience Inc., Holliston, MA, USA) was used throughout our experiment. Patch pipettes were filled with a cesium-based intracellular solution that contains the following (in millimolar): 115 cesium methanesulfonate, 10 cesium chloride, 10 4-(2-hydroxyethyl)-1-piperazineethanesulfonic acid, 10 tetraethylammonium chloride, 1 ethylene glycol-bis(β-aminoethyl ether)-N,N,N′,N′-tetraacetic acid, 3 adenosine triphosphate magnesium, and 0.5 guanosine 5′-triphosphate sodium salt hydrate, 2.0 mM L-glutathione reduced, 0.375-mM Fluo-4FF (Invitrogen, Carlsbad, USA), and 0.35-mM Cy3-labeled Ribeye-binding peptide (AnaSpec, San Jose, USA), pH 7.20, 290 mOsm. Pipette resistance is 4–6 MΩ range, and cells with a holding current exceeding −40 pA at −80 mV were excluded from the analysis. All patch-clamp experiments were carried out at room temperature, and the liquid junction potential was corrected offline.

Images of IHCs were taken with a two-photon microscope system (Scientifica Ltd., Uckfield, UK) using a 60× water immersion objective (Olympus, Tokyo, Japan). Cells loaded with Ca^2+^-indicator F4-FF and Cy3-conjugated peptide were excited by ultrafast pulsed titanium–sapphire laser (Coherent Inc., Santa Clara, USA) of 740 nm wavelength. The intracellular Ca^2+^ signal associated with IHC depolarization was acquired by using two-photon line scans (1.0 kHz) across the center of the fluorescent-labeled ribbon. The decay time of Ca^2+^ current transients was measured by fitting the calcium fluorescence decay with the following equation to assess the kinetic properties of Ca^2+^ extrusion.

$$\begin{array}{l} F=F_{0}+A1exp\left\{\frac{-\left(t-x_{t}\right)}{\tau 1}\right\}+A2exp\left\{\frac{-\left(t-t_{0}\right)}{\tau 2}\right\} \end{array}$$

where *F*_0_ is the initial luminescence intensity, *A1* and *A2* are pre-exponential factors, and (*t*–*t*_0_) is the difference between the initial time of measurement after excitation pulse *t*_0_ and time *t*. τ1 and τ2 is the fast and slow decaying component, respectively.

### Immunofluorescence Staining and Confocal Imaging

The dissected cochleae were perfused with 4% paraformaldehyde at 4°C overnight. The next day, the organ of Corti was dissected, then permeabilized with 5% Triton X-100 and blocked in 5% bovine serum albumin. Primary antibodies used in this experiment were mouse anti-CtBP2 immunoglobulin (Ig) G1 (BD Biosciences, Franklin Lakes, NJ, USA), mouse anti-GluR2 IgG2a (Merck-Millipore, Darmstadt, Germany), rabbit anti-calretinin (Abcam, Cambridge, UK), and rabbit anti-PNPase (Proteintech, Rosemont, IL, USA). The secondary antibodies used were Alexa Fluor 568-conjugated goat anti-mouse IgG1, Alexa Fluor 647-conjugated IgG2, and Alexa Fluor 488-conjugated goat anti-rabbit IgG (Invitrogen, USA). Confocal images were acquired on a Zeiss confocal microscope (Carl Zeiss Microscopy GmbH, Jena, Germany). Images were acquired under a Zeiss LSM 880 with a 63×, 1.4 numerical aperture oil objective lens. Pixel size was 0.1 μm in x and y planes and 0.38 μm for the z-axis. For synapse counts, the confocal stacks were created using 3D analysis software (Carl Zeiss Microscopy GmbH, Jena, Germany).

### Protein Extraction and Western Blotting

Animals were killed, and cochleae were dissected in ice-cold phosphate-buffered saline. For each sample, tissues from 6 Organs of Corti were collected and pooled, treated with ice-cold radioimmunoprecipitation assay lysis buffer plus protease inhibitor cocktail (Thermo Fisher Scientific, Waltham, MA, USA) and phosphatase inhibitors. The samples were then centrifuged at 10,000 × g at 4°C for 10 min. The supernatants were collected, and the protein concentration was determined by using a BCA Protein Assay Kit (Beyotime Institute of Biotechnology, Jiangsu, China) for protein quantification. Equal amounts of proteins were loaded onto a 10–12% sodium dodecyl sulfate-polyacrylamide gel electrophoresis gel, and after electrophoresis, proteins were transferred onto polyvinylidene difluoride membranes (Merck-Millipore). After blocking with 5% non-fat milk for 1 h at room temperature, the membranes were incubated with anti-cleaved caspase-3, anti-Bcl2, anti-Bax, anti-cytochrome C (Cell Signaling Technology, Danvers, MA, USA), anti-calpain 1/2, anti-calpastatin (Abcam), and anti-GAPDH (Beyotime) overnight at 4°C, with gentle shaking. The next day, the membrane was washed three times (10 min each) with phosphate-buffered saline with Tween 20 buffer and then incubated with an appropriate secondary antibody labeled with horseradish peroxidase (Abcam) for 1 h at 37°C. After extensive washing of the membrane, the protein bands were visualized using an Amersham Imager 600 (G.E. Healthcare, Little Chalfont, UK) using the elevated chemiluminescence procedure. Image J software (National Institutes of Health, Bethesda, MD, USA) was used to calculate the relative densities of the probe protein.

### Data Analysis and Statistical Tests

Data analysis was performed by using software Igor Pro (WaveMetrics Inc., Lake Oswego, OR, USA) and GraphPad Prism (GraphPad Software Inc., La Jolla, CA, USA). For comparisons between two samples, a two-tailed, unpaired Student's *t*-test or Mann–Whitney test was used. For multiple group comparison, statistical significance was calculated using one- or two-way ANOVA followed by Bonferroni *post-hoc* test. Cumulative distributions were statistically compared using the Kolmogorov–Smirnov test. Data are presented as mean ± SEM, and values of *P* < 0.05 were considered statistically significant. In figures, N.S. indicates that *P* > 0.05, ^*^indicates that *P* < 0.05, ^**^indicates that *P* < 0.01, and ^***^indicates that *P* < 0.001.

## Results

### CBA/CaJ Mice Were More Susceptible to Noise-Induced Synaptopathy

**Figure 1:**
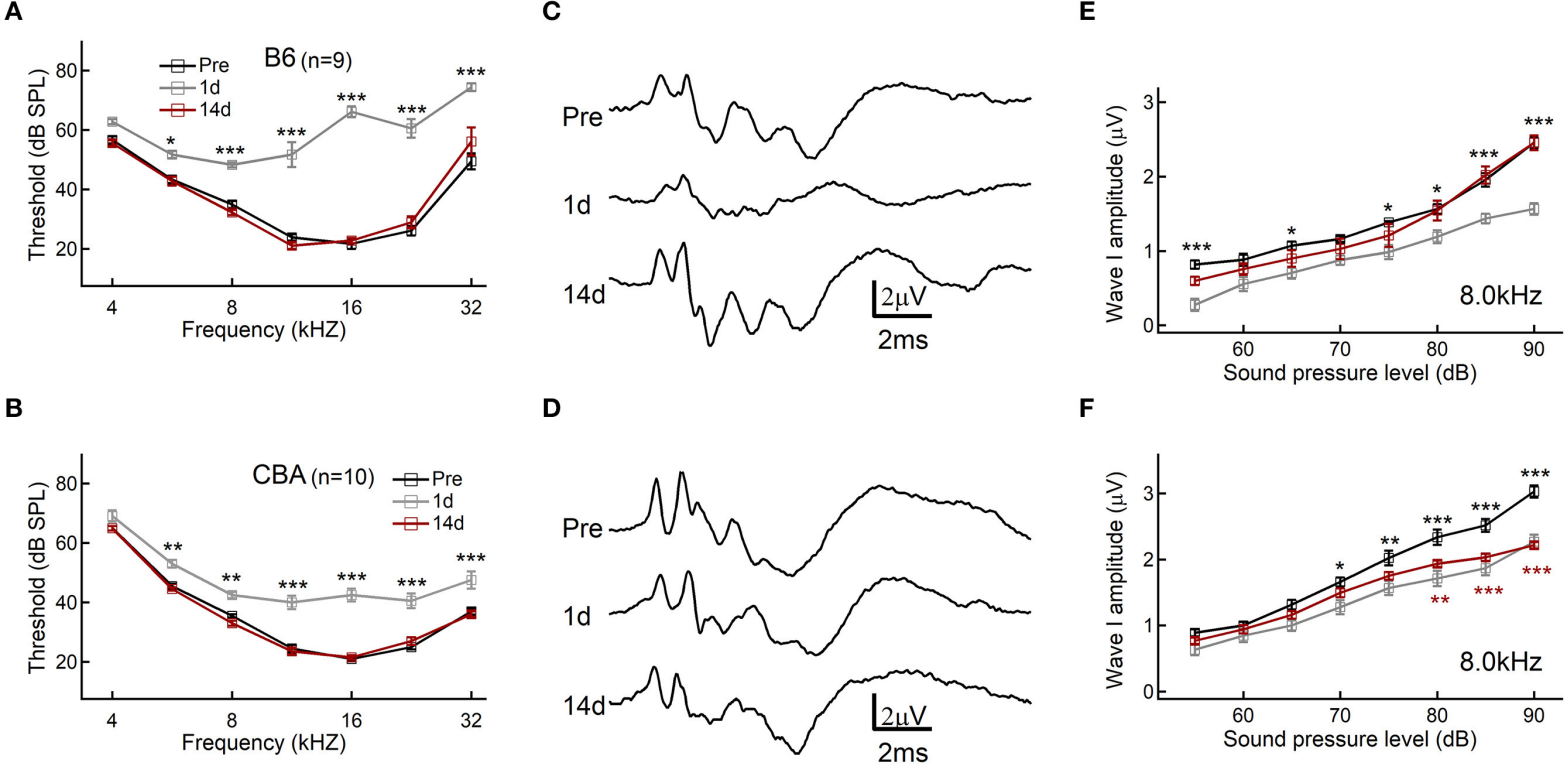
Auditory brainstem response (ABR) threshold and wave I analysis. **(A,B)** Hearing threshold of ABR before and after noise exposure. **(C,D)** Representative traces of ABR response to 80 dB SPL at 8.0 kHz. **(E,F)** ABR wave I amplitudes, evoked by suprathreshold tones at 8.0 kHz. Data are presented as mean ± SEM; statistical significance was assessed by two-way ANOVA followed by the Bonferroni *post-hoc* test, *P < 0.05, **P < 0.01, and ***P < 0.001.

To determine the hearing threshold shift and synaptopathy induced by acoustic exposure, we analyzed ABR recordings before and after noise exposure of B6 and CBA mice. ABR threshold shifts were similar, and temporal threshold shifts were observed in both B6 and CBA mice, which were both significantly elevated at 1 day [*F*_(1, 56)_ = 574, *p* < 0.0001; *F*_(1, 63)_ = 166.9, *p* < 0.0001, for B6 and CBA mice, respectively, *n* = 9 and 10, two-way ANOVA] and progressively recovered at 14 days after noise exposure [*F*_(1, 56)_ = 0.27, *p* = 0.61; *F*_(1, 63)_, *p* = 0.7606, two-way ANOVA, Figures 1A,B]. The first wave of ABR (Wave I) represents the summated activity of responding auditory afferent fibers (Figures 1C,D). Analysis of ABR wave I amplitude revealed that CBA mice exhibited a significant reduction of wave I amplitude reduction at 14 days after noise exposure at high SPLs starting from 80 dB (2.34 ± 0.11 vs. 1.93 ± 0.05 μV, *n* = 10 for both groups, two-way ANOVA followed by Bonferroni *post-hoc* test, *p* < 0.01, Figure 1F), whereas B6 wave I amplitude fully recovered (2.46 ± 0.07 vs. 2.45 ± 0.10 μV at 90 dB SPL, *n* = 9 for both groups, two-way ANOVA followed by Bonferroni *post-hoc* test, *p* > 0.05, Figure 1E). Because wave I of ABR is generated by the synchronous firing of auditory nerve fibers, these results indicate that CBA mice's IHC-SGN synaptic functions were more vulnerable to acoustic overexposure. To verify, we then quantify the changes in the synaptic count.

### Ribbon Synapse Number Was Incompletely Recovered in CBA/CaJ Mice at 14 Days After Noise Exposure

**Figure 2:**
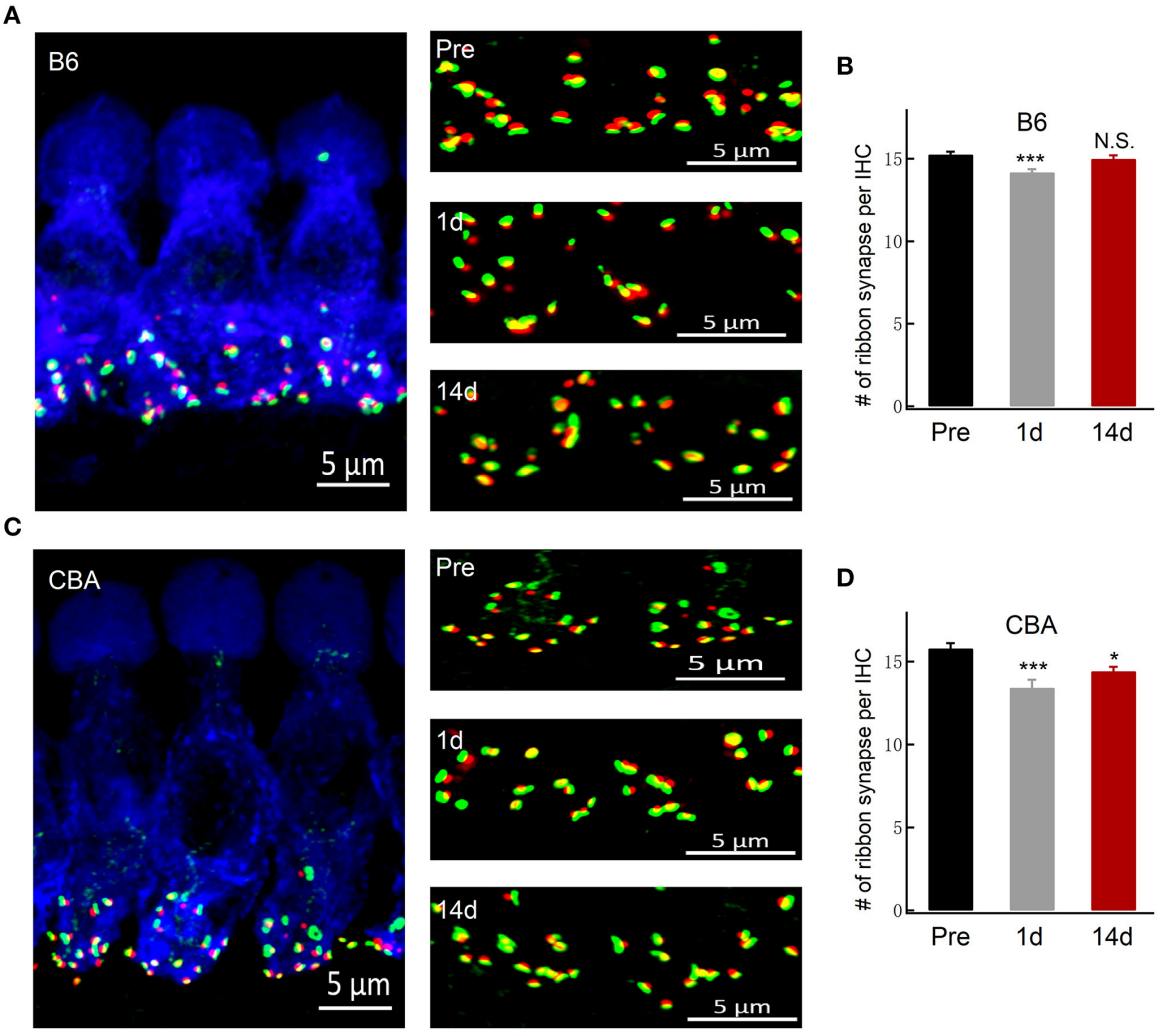
Inner hair cell ribbon synapse counts in B6 and CBA mice before and after noise exposure. **(A,C)** Representative confocal images of IHCs co-labeled for the presynaptic marker CtBP2 (red) and postsynaptic marker GluA2 (green) of B6 and CBA mice at pre, 1 day, and 14 days after noise exposure in the apical turn of the basilar membrane. **(B,D)** Puncta of co-labeling are presumably ribbon synapses, and the numbers of synapses were counted (mean ± SEM) per IHC. Data were analyzed by one-way ANOVA followed by Bonferroni *post-hoc* test. *P < 0.05 and ***P < 0.001. N.S., no significant difference was found.

A functional ribbon synapse consists of a presynaptic ribbon and postsynaptic AMPA receptors (Kim et al., 2019). Excessive noise exposure may lead to excitotoxicity that could damage the synapses. By co-staining the presynaptic ribbons (anti-CtBP2) and postsynaptic AMPA-type glutamate receptors (anti-GluA2) of the organ of Corti before and after noise exposure, we show in B6 mice that the number of ribbon synapses was initially reduced at day 1 after noise exposure but then recovered and indistinguishable from the pre-exposure level at day 14 (15.3 ± 0.16, 14.2 ± 0.19, and 15.0 ± 0.20; *n* = 31, 28, and 24; respectively, of pre, 1 day, and 14 days) after noise exposure (Figures 2A,B), in agreement with the *in vivo* ABR measurement of wave I amplitude measurement. On the other hand, for CBA mice, there was an incomplete recovery of the ribbon synapses after the noise exposure (15.8 ± 0.32, 13.4 ± 0.47, and 14.2 ± 0.47; *n* = 34, 19, and 26; respectively, of pre, 1 day, and 14 days), which support the finding of significant and lasting ABR wave I reduction (Figures 2C,D).

### Protein of Bcl-2/Bax Ratio and Cytochrome-C Was Expressed Higher in CBA/CaJ Mice After Noise Exposure

**Figure 3:**
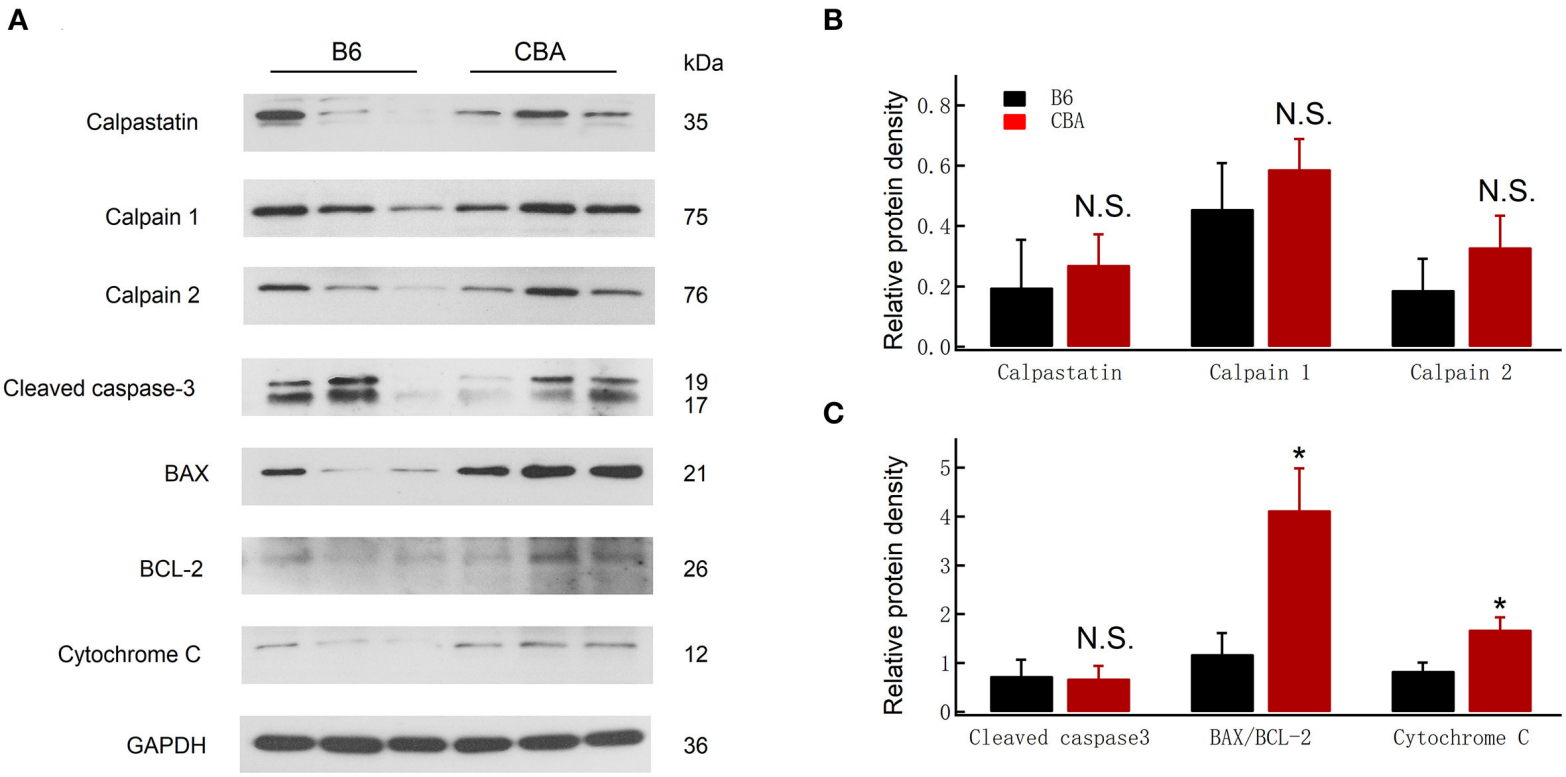
Comparison of protein expression at 1 day after noise exposure between CBA and B6. **(A)** Protein levels of cleaved caspase-3, Bax, Bcl 2, cytochrome-C, calpain 1/2, and calpastatin were detected by Western blotting; GAPDH was used as an internal control. **(B,C)** Relative protein levels are presented as relative ratio of target proteins to GAPDH. Data were analyzed by one-way ANOVA followed by Bonferroni *post-hoc* test. *P < 0.05. N.S., no significant difference was found.

Alterations in calcium homeostasis are widely reported to contribute to synaptic degeneration, and the Ca^2+^-dependent proteases play a causal role in axonal and synaptic degeneration (Ma et al., 2013). We therefore analyzed the protein levels of calpastatin (0.20 ± 0.16 vs. 0.27 ± 0.10, for B6 and CBA mice, respectively, unpaired *t*-test, *P* = 0.71), calpain 1 (0.46 ± 0.15 vs. 0.59 ± 0.10, for B6 and CBA mice, respectively, unpaired *t*-test, *P* = 0.50), and calpain 2 (0.19 ± 0.10 vs. 0.33 ± 0.10, for B6 and CBA mice, respectively, unpaired *t*-test, *P* = 0.39), but no significant differences were found between the two mouse strains (Figures 3A,B).

Free radicals and oxidative stress also play essential roles in the pathogenesis of NIHL (Liu et al., 2016, 2019c; He et al., 2017; Li et al., 2018b; Chen et al., 2020). Cytochrome-c is a biomarker of oxidative stress, which serves as an indicator of oxidative damage (Yamasoba et al., 2005). The activation of caspase-3, known to trigger widespread damage and degeneration, can also modulate synaptic function (Imbriani et al., 2019). Likewise, Bcl-2 expression is reduced, whereas Bax is increased in IHCs after noise exposure (Chen et al., 2020). Therefore, we investigated the expression of mitochondrial-dependent damage markers, such as Bax and cytochrome-c. As shown in Figure 3C, an increase in the ratio of Bax/Bcl-2 (1.19 ± 0.42 vs. 4.13 ± 0.85, for B6 and CBA mice, respectively, unpaired *t-*test, *P* = 0.036) and cytochrome-c release (0.85 ± 0.16 vs. 1.69 ± 0.25, for B6 and CBA mice, respectively, unpaired *t*-test, *P* = 0.047) was found in CBA mice after noise exposure. Meanwhile, the expression of cleaved caspase-3 was similar between the two strains. Taken together, these results revealed that noise-induced mitochondrial damage might be associated with the greater susceptibility to cochlear synaptopathy of CBA mice.

### Cytosolic Calcium Clearance at the Active Zone Rate Is Slower in CBA/CaJ mice

**Figure 4:**
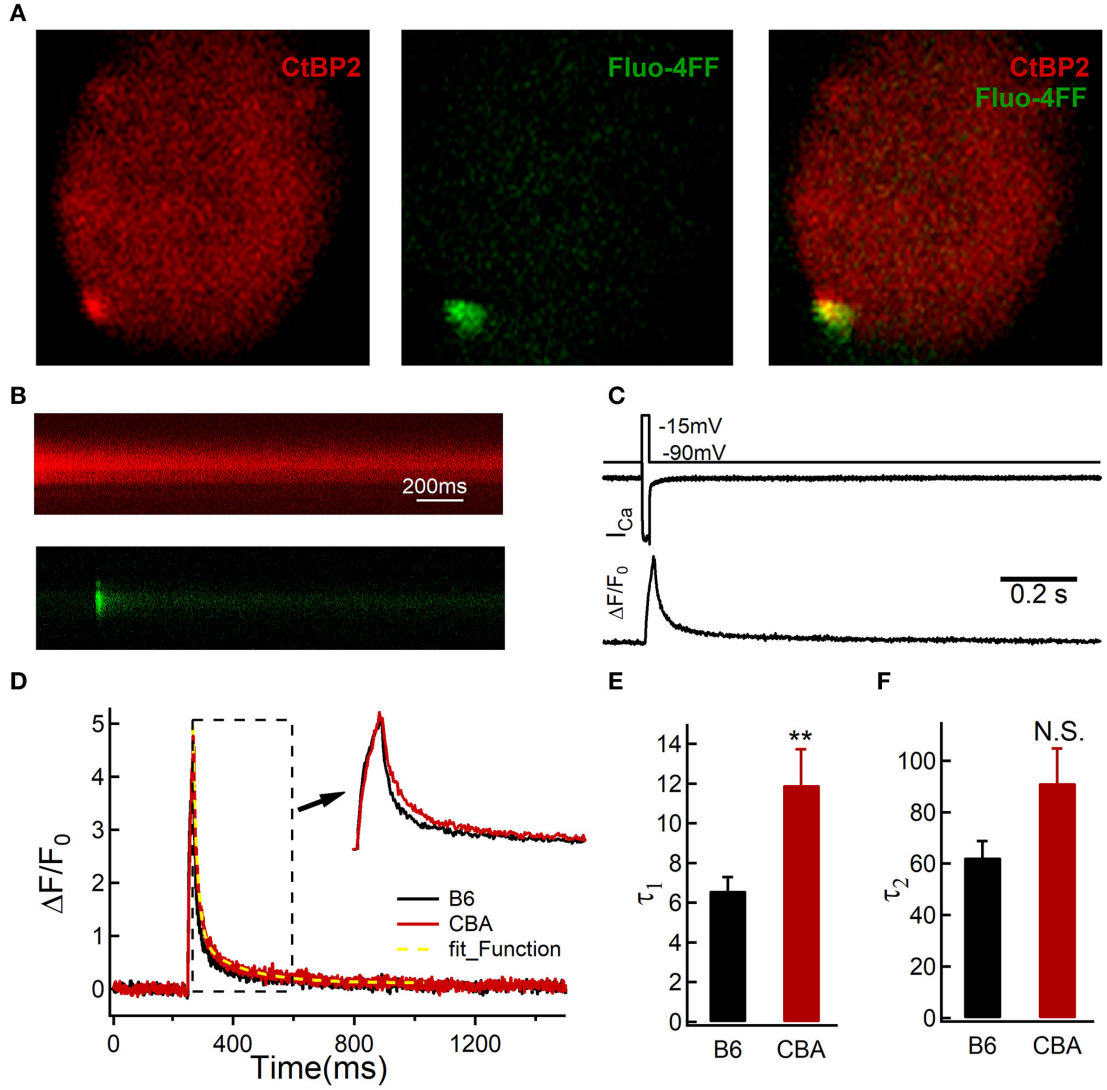
Two-photon calcium imaging in IHCs from the apical turn of the organ of Corti without noise exposure. **(A)** IHCs were loaded with Cy3-conjugated peptide binding to the synaptic ribbon to visualize synaptic ribbons at the active zone, and the hotspots of depolarization-evoked Ca^2+^ influx, visualized by increased fluorescence of the Ca^2+^ indicator Fluo-4FF. **(B)** Example line scan of a fluorescently-labeled ribbon and Fluo-4FF fluorescence change at an individual IHC active zone during 20-ms depolarization. **(C)** Representative recording show the evoked whole-cell Ca^2+^ current and the depolarization-evoked increase in fluorescence at a single active zone; ΔF from line-scans was normalized to their baseline fluorescence F_0_ hence ΔF/F_0_. **(D–F)** Decay of Ca^2+^ fluorescence intensity was fitted by a double exponential. Statistical significance was assessed by the Mann–Whitney test, **P < 0.01. N.S., no significant difference was found.

During noise exposure, the sustained Ca^2+^ entry could induce mitochondrial dysfunction (Chen et al., 2018). Cytosolic Ca^2+^ homeostasis is maintained through a delicate balance between the influx and efflux of calcium ions. To probe the reasons for the different outcomes after noise exposure, we further analyzed Ca^2+^ dynamics at the single active zone level using two-photon Ca^2+^ imaging (Wong et al., 2014). We loaded IHCs with a Cy3-conjugated Ribeye-binding peptide to locate ribbons (excitation wavelength: 740 nm) and the low-affinity Ca^2+^ indicator Fluo-4FF (*K*_*d*_ = 9.7 μM, excitation wavelength: 740 nm; Figure 4A). A line scan across the center of fluorescently-labeled ribbons during IHC depolarization (20 ms to −14 mV, 1 kHz) recorded the fluorescence change of Fluo-4FF (Figures 4B–D) (Krinner et al., 2017). The time constant fitted by a double exponential function during the decay phase of the Ca^2+^ transient represents the rate of cytosolic Ca^2+^ clearance (Kim et al., 2003; Chamberland et al., 2019). Although the slow-decaying component was similar between the two mouse strains (62.45 ± 26.34 vs. 91.30 ± 49.87 ms, for B6 and CBA mice, respectively, *n* = 17 and 14; Mann–Whitney test, *P* = 0.08; Figure 4F), IHCs of B6 mice that had a shorter fast decay time constant (6.62 ± 2.78 vs. 11.93 ± 6.70 ms, for B6 and CBA mice, respectively, *n* = 17 and 14; Mann–Whitney test, *P* = 0.001; Figure 4E), suggest that the Ca^2+^ clearance capability of the IHCs was more efficient in B6 mice.

### Mitochondria Around a Presynaptic Ribbon and the Expression of Calretinin in Inner Hair Cells Showed No Difference Between C57BL/6J and CBA/CaJ Mice

**Figure 5:**
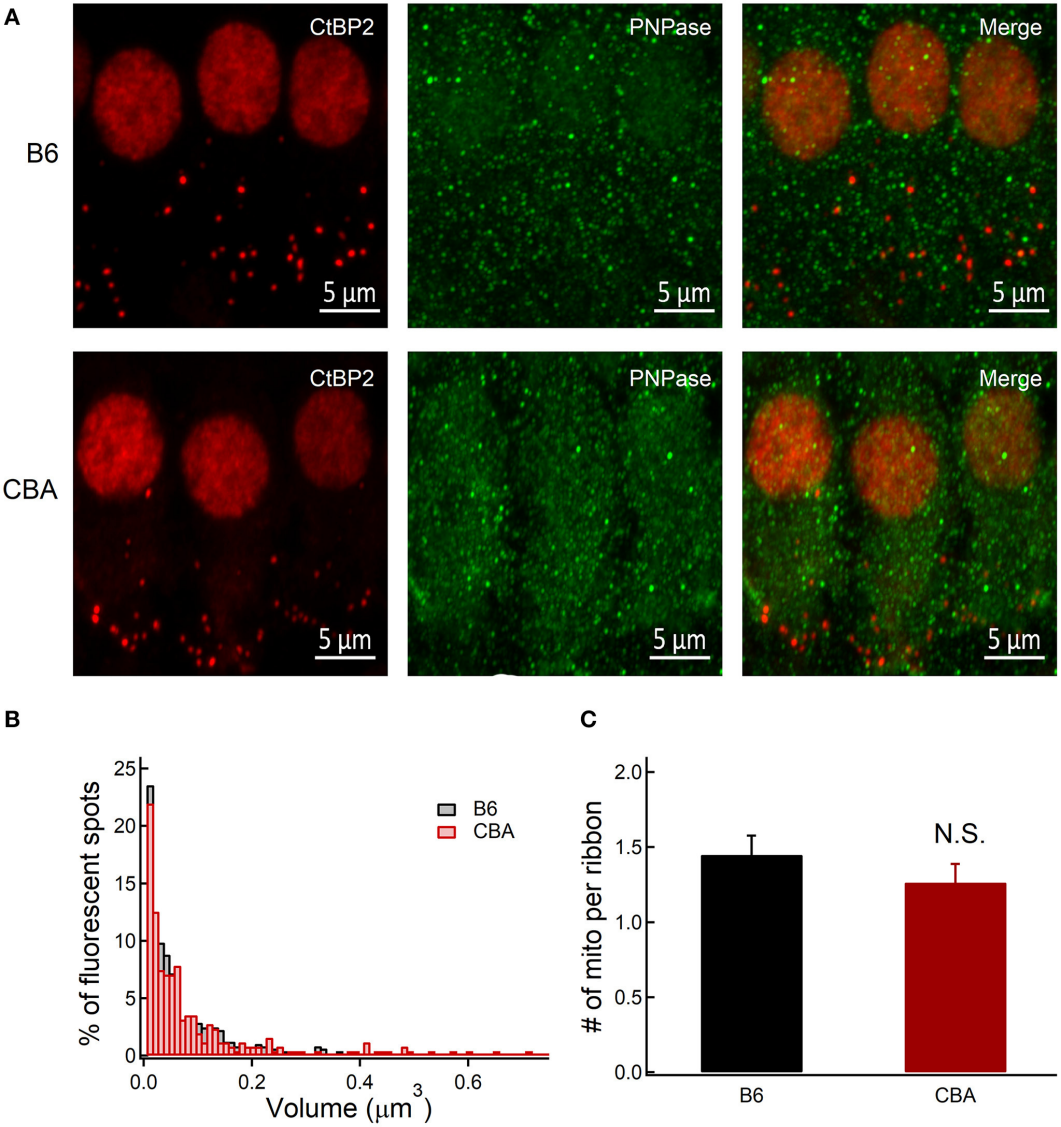
Immunofluorescence analysis of the presynaptic abundance of mitochondria counts between CBA and B6 mice without noise exposure. **(A)** Organ of Corti confocal image stacks of B6 and CBA mice, stained for CtBP2 and PNPase, show similar distribution in IHC at the apical turn of basilar membrane. **(B)** Normalized distributions of volumes of confocal z-sections integrated within a 0.5-μm radius around the center of mass of CtBP2 fluorescence in single confocal sections show near-identical distribution pattern. **(C)** Number of mitochondria within a 0.5-μm radius around the center of mass of CtBP2. Statistical significance was assessed by two-sample Kolmogorov–Smirnov test and unpaired *t-*test. N.S., no significant difference was found.

Mitochondrial calcium overload has been postulated to regulate a wide range of processes involved in NIHL (Wang et al., 2018). The mitochondrial calcium uniporter (MCU) is a major specific calcium channel for calcium uptake, and excessive cellular Ca^2+^ can rapidly enter the mitochondria through the MCU (Rizzuto et al., 2012). Therefore, we compared the volume with the number of mitochondrial around the presynaptic ribbons (Figure 5). Presynaptic mitochondrial volumes (integrated within a 0.5-μm radius around the center of mass of CtBP2 fluorescence in single confocal sections) were calculated in a total of 488 and 255 regions of six mice's organ of Corti in each group. There was no difference in the distributions of mitochondrial volumes around the presynaptic ribbon between the two animal strains (*D* = 0.05, two-sample Kolmogorov–Smirnov test, *P* = 0.76; Figure 5B). In addition, similar results were observed with regard to the number of mitochondria per ribbon within the region (1.45 ± 0.13 *vs*. 1.26 ± 0.12, for B6 and CBA mice, respectively, unpaired *t*-test, *P* = 0.37; Figure 5C).

**Figure 6:**
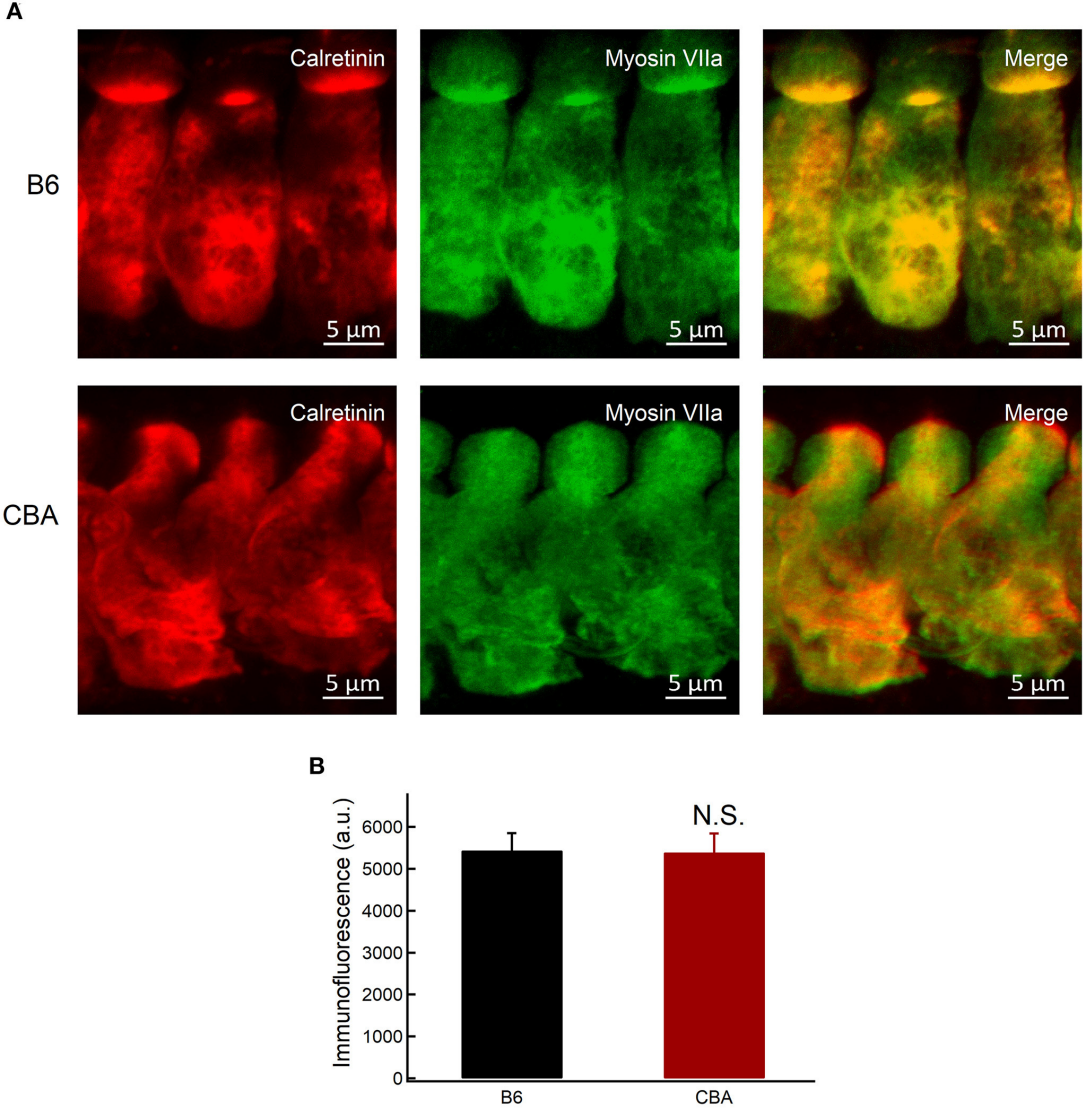
Expression of calretinin in IHCs. **(A)** Whole-mount preparation of organ of Corti double-stained for calretinin and myosin VIIa. **(B)** Quantification of calretinin fluorescence at the base of IHCs in apical region without noise exposure. Statistical significance was assessed by unpaired *t*-test. N.S., no significant difference.

Neuroprotective Ca^2+^-buffering proteins have been proposed to be related to protection against traumatic noise exposure (Alvarado et al., 2016). Calretinin is a Ca^2+^-buffering protein with neuroprotective action (D'Orlando et al., 2002), and the absence of calretinin might be associated with the noise susceptibility of the fibers (Sharma et al., 2018). The expression of calretinin was measured by immunofluorescence quantification. Fluorescence intensity was averaged in Gaussian volumes with standard deviations of 1 μm along the X, Y, and Z axes (Michalski et al., 2017), centered around points selected at the base of IHCs of each confocal stack (for a total of 39 B6 IHCs and 32 CBA IHCs in six mice of each group). We showed that the intensity of calretinin was comparable between the two groups (5.443 ± 412 vs. 5.397 ± 450.4, for B6 and CBA mice, respectively, unpaired *t*-test, *P* = 0.66; Figures 6A,B). Together with the findings mentioned earlier, we hypothesized that mitochondria in IHCs of CBA mice might take up more Ca^2+^ under noise exposure and result in mitochondrial Ca^2+^ overload, resulting in susceptibility to noise-induced synaptopathy.

## Discussion

Intense HC stimulation will cause damage to IHC ribbons, as well as to postsynaptic receptors (Kujawa and Liberman, 2009), which has been implicated as a contributor to noise-induced hearing impairment (Henry and Mulroy, 1995), eventually induce apoptotic cell death in HCs, especially the outer HCs of the basal turn (Sun et al., 2014; He et al., 2016; Yu et al., 2017; Li et al., 2018a; Zhang et al., 2019). CBA and B6 mice have been used extensively to explore mechanisms underlying hearing loss for many years (Li and Hultcrantz, 1994; Spongr et al., 1997). B6 mice carry a specific mutation in cadherin23 (Cdh23), which lead to disorganized hair bundles (Noben-Trauth et al., 2003), rendering the mice more susceptible to noise insult (Davis et al., 2001). However, these two animal models exhibited different susceptibilities to noise-induced synaptopathy, in that the ribbon synapse in B6 mice can completely recover (Shi et al., 2015), while not the case in CBA mice. Few studies have been published to focus on this difference and explore the possible mechanisms. Our results identified candidate mechanisms underlying the susceptibility to noise-induced synaptopathy and provide guidance in animal model selection.

As reported in a previous study (Kujawa and Liberman, 2009), we found that CBA mice showed the typical feature of “hidden hearing loss,” in which a temporal threshold shift was detected by ABR and a decreased ABR wave I amplitude was observed. Meanwhile, in B6 mice, the ABR wave I was completely recovered, which was consistent with previously published studies (Kim et al., 2019). The repair/regeneration of ribbon synapses, which could present as changes in ABR wave I amplitude (Kujawa and Liberman, 2009), may depend on animal species, experimental conditions, and the actions of neurotrophic factors after acoustic exposure (Shi et al., 2013; Kim et al., 2019). Thus, we concluded that the IHCs of the two strains underwent different cellular/subcellular changes in response to noise exposure.

Calcium channels in the IHC presynaptic active zones are key signaling elements that transform sound-evoked presynaptic potential into neurotransmitter release. Ca^2+^ homeostasis of the sensory HCs is particularly critical for presynaptic electrical activities, synaptic transmission, and efferent modulation (Lenzi and Roberts, 1994). Prolonged alterations of intracellular calcium have been shown to cause neuronal excitotoxicity (Arundine and Tymianski, 2004). During noise exposure, more voltage-gated Ca^2+^ channels continue to open, causing sustained Ca^2+^ entry into IHCs from the extracellular space and elevated the intracellular free Ca^2+^ ions, which has been implicated in a variety of pathological conditions (Brookes et al., 2004). Under these circumstances, the capability of modifying intracellular Ca^2+^ homeostasis appeared to be crucial in maintaining IHC function. Dysfunction of Ca^2+^ extrusion channels can lead to intracellular Ca^2+^ overloading and eventually cell death or neurodegeneration (Hajieva et al., 2018). Thus, Ca^2+^ clearance may play an additional role in neurodegenerative conditions.

The elevation of cytosolic Ca^2+^ alone may not be a major contributing factor to HC death, but the dysfunction of mitochondria is likely the defining event (Esterberg et al., 2014). Low levels of mitochondrial Ca^2+^ uptake can feed energetically active cells through ATP production; however, prolonged uptake can be toxic, leading to an increase in the production of reactive oxygen species production and the release of cytochrome c (Giorgi et al., 2008, 2012). The excessive cytosolic Ca^2+^ uptake by mitochondria through the Ca^2+^ uniporter could lead to mitochondrial dysfunction, which has been implicated in synaptopathy and can contribute to neurodegeneration (Flippo and Strack, 2017). However, the inhibition of MCU activity could attenuate noise-induced loss of sensory HCs, synaptic ribbons, and ABR wave I amplitudes (Wang et al., 2018). Our results found a slower calcium clearance rate and the same levels of Ca^2+^ buffering proteins, which indicated that the mitochondrial might uptake more Ca^2+^. Taken together, we presented a mechanism that presynaptic- and mito-Ca^2+^ couple to induce the cochlea synaptopathy.

In summary, we demonstrated temporary and persistent alterations of ABR wave I and ribbon synapses in B6 and CBA mice, respectively, and CBA mice showed more susceptibility to noise-induced synaptopathy between the IHCs and SGN. This may be accounted for by the elevated Ca^2+^ around the ribbon and further leads to mitochondrial dysfunction under noise exposure in CBA mice. Further research is required to clarify the possible mechanism between calcium clearance and presynaptic dysfunction in noise-induced synaptopathy.

## Data Availability Statement

The original contributions presented in the study are included in the article/supplementary material, further inquiries can be directed to the corresponding author/s.

## Ethics Statement

The animal study was reviewed and approved by University Committee of Laboratory Animals of Shanghai 9th people's hospital.

## Author Contributions

HuL, ZW, and HW conceived and designed the research. HoL, LW, and PX conducted the experiments. HoL and HP analyzed the generated data and wrote the manuscript. All authors have read and agreed to the published version of the manuscript.

## Conflict of Interest

The authors declare that the research was conducted in the absence of any commercial or financial relationships that could be construed as a potential conflict of interest.
